# Use of a static progressive stretch orthosis to treat post-traumatic ankle stiffness

**DOI:** 10.1186/1756-0500-5-348

**Published:** 2012-07-04

**Authors:** Christopher R Costa, Mark J McElroy, Aaron J Johnson, Bradley M Lamm, Michael A Mont

**Affiliations:** 1 Center for Joint Preservation and Replacement at the Rubin Institute for Advanced Orthopedics, Baltimore, Maryland, USA; 2 International Center for Limb Lengthening at the Rubin Institute for Advanced Orthopedics, Baltimore, Maryland, USA; 3 Rubin Institute for Advanced Orthopedics, Center for Joint Preservation and Reconstruction Sinai Hospital of Baltimore, 2401 West Belvedere Avenue, Baltimore, Maryland, 21215, USA

**Keywords:** Ankle, Stiffness, Orthosis, Progressive stress relaxation, Rehabilitation

## Abstract

**Background:**

Chronic ankle stiffness can develop for numerous reasons after traumatic injury, and may adversely affect patient gait, mobility, and function. Although standard physical therapeutic techniques typically resolve this stiffness, some cases may be recalcitrant to these measures, making it difficult to restore range-of-motion. The purpose of this study was to evaluate a static progressive stretch orthosis for the treatment of chronic ankle stiffness.

**Methods:**

Twenty-six patients (26 ankles) who had chronic post-traumatic ankle stiffness were studied. The patients began treatment at a mean of 47 weeks (range, 6 to 272 weeks) following their initial injury using a static progressive stretch orthosis. A patient-directed protocol was used for 30 minutes per day, 1 to 3 times per day, until the range-of-motion was considered to have plateaued. Mean treatment time was 10 weeks (range, 3 to 19 weeks). Treatment duration, range-of-motion, and complications with the device were assessed.

**Results:**

The overall mean improvement in motion (combined dorsiflexion and plantar flexion) was 17 degrees (range, 2 to 44 degrees). There was a mean improvement in dorsiflexion of 9 degrees (range, -2 to 20 degrees), and a mean improvement of 8 degrees of plantar flexion (range, -10 to 35 degrees). There were no reports of numbness or skin problems.

**Conclusions:**

The outcomes of this study suggest that a patient-directed treatment protocol using a static progressive stretch orthosis was an effective ancillary method for the treatment of chronic post-traumatic ankle stiffness that was refractory to standard physical therapy techniques.

## Background

Stiffness of the tibio-talar joint may occur following fracture, stroke, or prolonged immobilization, which may affect patient gait and mobility. Rehabilitation treatment protocols have been developed for patients after adverse events in order to treat this problem or to avoid it entirely. However, despite these measures, sometimes ankle stiffness persists and the only treatment option that exists may be surgical intervention. [1,2] Strategies for improving patient function and avoiding additional surgery for stiff joints include passive and patient-controlled active stretching devices [3-8], which have been shown to improve range-of-motion and increase tendon flexibility.

Dynamic ankle-foot-orthosis brace designs work by increasing the range-of-motion of the joint by applying a constant, low-load, passive stress in dorsiflexion or plantar flexion. Although there are a variety of devices that may accomplish this, the theoretical basis behind their designs is the same: over time, the applied forces result in remodeling of the surrounding tissue allowing for increased laxity and tendon length, thus increasing range-of-motion. [9] However, it can take several months to see improvements and loss of flexibility may occur once the brace is removed. [10-12] One reason for variable success rates may be due to low compliance with these devices. These braces are designed to be worn for up to 12 hours a day, which may be uncomfortable, and may lead to skin breakdown. Static progressive stretch devices need only be worn for 30 several minute sessions per day, and are patient-directed, which may allow patients to shorten their treatment duration if they are able or willing to tolerate higher levels of discomfort during the treatment sessions.

Alternatively, braces have been developed that improve range-of-motion through intervals of stress and relaxation as opposed to the static passive models. These devices work by applying a constant force to the joint in dorsi- or plantar flexion and then gradually increasing that force over a short period of time (every 5 to 10 minutes) after the tissue accommodates to the new load. This dynamic applied stress allows stretching and remodeling of the surrounding tissue and ligaments to occur at an accelerated rate compared to passive stress devices [13-15]. The potential advantage of these devices is that they do not have to be worn as many hours on a daily basis and they may accelerate rehabilitation. However, currently there is a paucity of literature examining the results of these devices for ankle stiffness in a clinical setting outside of traumatic brain injury [16].

The purpose of this study was to evaluate a static progressive stretch orthosis for the treatment of patients with post-traumatic ankle stiffness. Clinical outcomes were assessed by improvements in active dorsiflexion and plantar flexion and overall range-of-motion after a standardized patient directed therapy protocol. Also, we evaluated the incidence of any complications that occurred during the use of this orthosis.

## Methods

Twenty-six consecutive patients (26 ankles) who had chronic post-traumatic ankle stiffness refractory to standard physical therapy were retrospectively reviewed from data collected in a prospective database. Chronic post-traumatic ankle stiffness was defined as loss of either plantar flexion or dorsiflexion by more than 5 degrees in patients who did not undergo treatment with a static progressive stretch orthosis device for a minimum of 6 weeks after their initial injury; the normal range of motion of the ankle is taken to be 0 to 50 degrees of plantar flexion, and 0 to 20 degrees of dorsiflexion [17]. Patients were excluded if their primary diagnosis for stiffness was due to an atraumatic flexion contracture or if there was radiographic evidence of fusion of the tibio-talar joint or heterotopic ossification that might mechanically impinge on the range-of-motion of the joint. Institutional review board approval was obtained for the study of these patients.

There were 9 men and 17 women who had a mean age of 48 years (range, 20 to 77 years). Fifteen of the patients had suffered an injury of the ankle joint that required surgical intervention and eight patients had an ankle injury that was managed non-operatively. Three patients had ligamentous injuries; 2 of them required surgical intervention, and 1 was managed non-operatively. The mean time from initial injury to treatment was 47 weeks (range, 6 to 272 weeks). Twenty-four of the patients had undergone some degree of outpatient physical therapy, but still had refractory ankle stiffness. The mean time spent in physical therapy prior to enrollment in this study was 12 weeks (range, 2 to 39 weeks). The mean initial active dorsiflexion prior to treatment was -7 degrees (range, 15 to -30 degrees) and the mean initial active plantar flexion was 41 degrees (range, 20 to 60 degrees). Throughout the manuscript, negative values indicate a contracture of the described amount (i.e. an initial dorsiflexion of -7 degrees and initial plantarflexion of 15 degrees indicates that the patient is in equinus, with a total range of motion of 7 degrees of plantar flexion to 15 degrees of plantarflexion, for a total range-of-motion of 8 degrees).

**Figure 1:**
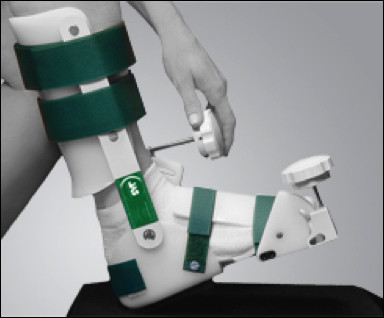
JAS Ankle orthosis (Joint Active Systems, Effingham, IL).

All patients were treated with the JAS™ Ankle orthosis (Joint Active Systems, Effingham, Illinois) pictured in Figure 1. The device is based on the principles of static progressive stretch and is operated by the patient. A knob adjusted by the patient can range the device from 55 degrees of dorsiflexion to 45 degrees of plantar flexion.

Patients were initially seen in the office for proper fitting of the brace and instructions on how to use the device. After all questions were answered, they were given detailed instructions and a phone number to call if they had any questions or problems with the device. The treatment protocol consisted of wearing the ankle orthosis for 30 minutes a day for a week. Patients were instructed to use the knob to advance dorsiflexion of the device until they felt tension, but not pain at the tibio-talar joint. They were then told to advance the knob every 5 minutes into further flexion until they felt the same tension. Patients with stiffness in both plantar flexion and dorsiflexion were instructed to perform the same 30 minute range-of-motion exercise after 15 minutes of rest from the previous dorsiflexion exercise. During the second week of treatment, they were instructed to perform the range-of-motion exercises twice a day. After 2 weeks, patients were instructed to perform the exercises 3 times a day for the duration of their therapy. The orthosis was used until gains in range-of-motion ceased to occur for five consecutive days. At intervals between therapy sessions, patients were instructed to take the brace off and could weight-bear and perform activities as tolerated. No specific restrictions were place upon the patients outside of the time they were in the brace.

Clinical outcomes were assessed by measurement of dorsi- and plantar flexion, with use of a goniometer by a patient-consistent physical therapist at regularly scheduled office appointments (approximately 2 times per week). After completion of brace use, patients were scheduled to follow-up at 3 months and at 1 year for re-evaluation.

During clinic visits, all patients were questioned about any complications they had experienced during the use of the brace, such as increased pain, numbness, decreased strength, or skin irritation. A physical examination was performed at all follow-up visits to assess for skin breakdown, pressure ulcers, or excessive joint laxity. The mean duration of treatment with the brace was 10 weeks (range, 3 to 19 weeks). No additional rehabilitation or physical therapy modalities were performed on the patients during this time period.

All data was collected and analyzed using a spreadsheet in SPSS Statistics 17.0,1 (SPSS Inc., Chicago, Illinois). Descriptive variables, such as mean, minimum, and maximum values, were calculated in SPSS. The null hypothesis that was tested in this study was that there was no difference in total range of motion after the use of a static progressive stretch ankle orthosis. A paired t-test was used to determine significance in improvement of range-of-motion for dorsi- and plantar flexion before and after therapy. Patients were stratified by the amount of time that had elapsed between their injury and the start of treatment with the static progressive stretch device, either less than or greater than 60 weeks. Additionally, range-of-motion improvements were stratified into dorsiflexion or plantar flexion improvements. In patients who had both dorsiflexion and plantar flexion stiffness, these results were reported separately. For total range of motion, these values were summed (e.g. combined improvement in dorsiflexion and plantar flexion). Data was determined to be significant if *p* < 0.05.

## Results

The overall improvement in range-of-motion was 17 degrees (combined dorsiflexion and plantar flexion) after completion of therapy (range, 2 to 44 degrees). The mean improvement in dorsiflexion was 10 degrees (range, -2 to 20 degrees), from a pre-treatment mean of -7 degrees (range, -30 to 15 degrees) to 3 degrees (range, -20 to 20 degrees) and was found to be statistically significant (*p* < 0.05). The mean improvement in plantar flexion was 8 degrees (range, -10 to 35 degrees), from 34 degrees (range, 10 to 50 degrees) to 42 degrees (range, 20 to 60 degrees), which was also statistically significant ( *p* <0.05).

When stratified by duration between injury and start of static progressive stretch therapy, there was a significant difference in the range-of-motion gains. Patients who started the static progressive stretch device within 60 weeks of their injury had a mean improvement in range-of-motion of 17 degrees (range, -2 to 27 degrees), and the patients who started 60 weeks or more after their injury had a mean improvement in range-of-motion of 6 degrees (range, 2 to 15 degrees) (*p* = 0.008). Of note, the one patient who had negative total gains (e.g. worse range-of-motion after treatment) reported not using the device. All other patients reported compliance of using the device for at least one 30-minute session per day.

There were no reports of complications during the use of this treatment modality. Patients did not report any incidence of numbness or decreased strength during or after use of the brace. Physical examination did not reveal any skin irritation or breakdown following the use of the orthosis.

## Discussion

Ankle contractures are often debilitating to patients and may result in pain, altered gait, or decreased overall activity. Historically, treatment to regain range-of-motion has involved intensive physical therapy for several times a week for many months. Additional therapy has evolved to include the use of custom orthosis brace devices, which are typically worn for up to 12 hours a day for several months. The goal of these interventions is to increase ankle range-of-motion and maintain it after therapy has been completed. Because of the long duration of therapy regimens that are often associated with ankle function rehabilitation, this study analyzed whether a new orthotic device that used graduated stretch and relaxation would be able to increase range-of-motion in patients with ankle stiffness secondary to trauma. The results of this study showed that, at a mean of 10 weeks, there was significant improvement of dorsi- and plantar flexion with a total brace time that had a mean of approximately 90 minutes per day (*p* < 0.05).

There were some limitations of the present case series. The number of patients who met the inclusion criteria for the study was relatively small (n = 26). These small numbers limited our ability to stratify the data to perform any statistical analysis of various demographic features. Another limitation was that there was no randomized matched control group who underwent therapy with traditional static orthoses with which to compare our results. However, the authors believe that the significant improvement in ankle range-of-motion over a relatively short time period in a group of patients who had plateaued in recovery and the paucity of literature on this subject make these results notable. Additionally, it should be noted that although the device was used to treat ankle “stiffness”, range-of-motion was used as an index for stiffness, and an improvement in range-of-motion may not necessarily indicated reduced joint stiffness. Nevertheless, future studies utilizing a randomized prospective design would be ideal to establish which bracing methods produce the best gains in range-of-motion over the shortest time period.

Little literature regarding the use of orthotic stretch devices to increase range-of-motion in patients with ankle stiffness. Similar studies have been performed in other articulating joints, such as the wrist, elbow, and knee [8,18-20]. Ulrich *et al.* performed a study analyzing the use of a static progressive stress relaxation brace in patients with elbow stiffness [8]. The study included 37 patients who had a mean age of 45 years (range, 22 to 78 years) and who used the device from 30 to 90 minutes a day. Range-of-motion increased a mean of 26 degrees (range, 2 to 60 degrees) after a mean of 10 weeks of use (range, 2 to 22 weeks). They noted that patients had less analgesic use after completion of using the brace. McGrath et al. reported on the use of a static progressive stress relaxation device for the treatment of stiffness in the wrist. Their study included 47 patients who used the device for 30 to 60 minutes, 1 to 3 times a day. They noted a mean increase in wrist arc motion of 35 degrees (range 5 to 100 degrees).

The use of this device offers another method to treat chronic ankle stiffness after physical therapy has failed. There are few studies in relation to methods to increase ankle range-of-motion, and most are not specifically related to post-traumatic stiffness. Other methods that have been used include botulinum toxin A injections and active weight-bearing stretch exercises, but these have been less successful. [21,22] Kay *et al*. performed a randomized prospective trial using botulinum toxin to treat ankle equinus contractures in addition to serial casting in 23 patients who had cerebral palsy. [22] They found that the addition of botulinum toxin did not speed recovery or improve results in patients with ankle contractures compared to casting alone, as the mean dorsiflexion was -6.4 + 8.3 degrees with botulinum toxin, and -3.7  + 8.7 degrees without botulinum toxin ( *p* = 0.264). Additionally, its use was associated with earlier recurrence of spasticity, equinus during gait, and muscle contracture. Radford et al. performed a systematic review of studies that examined static stretching exercises as a way to increase ankle range-of-motion. Their review identified 5 studies, for a total of 161 patients. Studies varied from 3 days to 6 weeks in duration. Results showed an increase of 2.1 to 3 degrees of range of motion between 5 to 60 minutes. The greatest increases in range of motion were noted between 15 to 30 minutes of stretching where the stretching group range of motion improved by 3 degrees compared to patients who did not stretch ( *p* = 0.03). They concluded that calf muscle stretching offered a small, but statistically significant increase in ankle dorsiflexion compared to not stretching. However, all of the studies included in this review were of healthy people who had not previously suffered any ankle trauma or stiffness.

## Conclusions

The outcomes of this study suggest that a patient-directed treatment protocol using a static progressive stretch orthosis was an effective method for the treatment of chronic post-traumatic ankle stiffness that was refractory to standard physical therapy. This may be a useful ancillary therapeutic modality for helping patients improve gait and mobility and allowing them to actively participate actively in their rehabilitation. Additionally, we urge practitioners to consider static progressive stretch treatment earlier in the treatment course, as those who were treated less than 50 months after their injury showed greater gains in motion when compared to those who were treated later.

## Competing interests

Michael A. Mont is a consultant for Stryker Orthopaedics, Wright Medical, Johnson & Johnson, and Sage Products, Inc.; receives royalties from Stryker; and receives research or institutional support from Stryker, Wright Medical, the National Institutes of Health (NIAMS and NICHD), Tissue Gene, and Sage Products, Inc. Aaron J. Johnson is a consultant for Sage Products, Inc. The remaining authors have nothing to disclose.

## Authors’ contributions

The concept and design of the study was carried out by MAM, CRC, and BML; data was aqcuired by CRC, AJJ, MJM; data analysis was performed by AJJ, CRC, MJM; the manuscript was prepared by CRC, AJJ, MAM, BML; and final approval for publication was provided by MAM and BML. All authors read and approved the final manuscript.

## Authors’ information

Data was collected from multiple physical therapy locations throughout the United States. Institutional Review Board approval was obtained from St. Anthony’s Memorial Hospital, Effingham, Illinois.
